# Combined effect of olfactory ensheathing cell (OEC) transplantation and glial cell line-derived neurotrophic factor (GDNF) intravitreal injection on optic nerve injury in rats

**Published:** 2010-12-28

**Authors:** Yong Liu, Zili Gong, Lan Liu, Hanjun Sun

**Affiliations:** 1 Department of Neurology, Xinqiao Hospital, Third Military Medical University, Chongqing, China; 2 Department of Ophthalmology, Xinqiao Hospital, Third Military Medical University, Chongqing, China

## Abstract

**Purpose:**

To investigate the combined effect of olfactory ensheathing cell (OEC) transplantation and recombinant human glial cell line-derived neurotrophic factor (rhGDNF) intravitreal injection on optic nerve functional recovery following incomplete injury in adult rats.

**Methods:**

The optic nerves of adult rats were crushed by forceps and then GDNF was injected into vitreous cavity, OECs transplanted into injured optic nerve, or GDNF vitreous injection combined with OECs transplantation, and balanced salt solution was injected into vitreous cavity of control group rats respectively. Flash visual evoked potential (F-VEP) was performed on the injured eye immediately after injury and at 1, 2, 4, and 8 weeks after injury.

**Results:**

The F-VEP waveforms were almost silent immediately after the optic nerve injury. The latency of the F-VEP (LP1) recovered nearly to the normal value 1 week after injury in the treatment groups. The amplitude recovered more slowly. It recovered more obviously and rapidly in the rhGDNF combined with OEC group. At 8 weeks after injury, the amplitude was restored to 64.5% of the pre-injury level in the control group and to 91.8% in the GDNF+OEC treatment group. Wheat germ agglutinin (WGA) labeling showed retinal ganglion cell (RGC) axon regeneration and prolongation in the combined group, and the regenerated axons extended across the traumatized area and reached the distal end of the injured optic nerve.

**Conclusions:**

The combination of OEC transplantation and rhGDNF intravitreal injection will be more effective in promoting the recovery of visual function after incomplete injury of the optic nerve in adult rats.

## Introduction

The optic nerve is part of the central nervous system and cannot easily regenerate after injury. Incomplete optic nerve injury occurs frequently [1,2] and it is thought that improving the microenvironment by means such as cell transplantation and administration of exogenous neurotrophic factors may promote the regeneration of injured optic nerve fibers [3,4]. Various therapeutic methods may act on the nervous tissue differently, and the combination of two methods may exert a better effect than one method alone. Therefore, combining multiple therapeutic methods may become the main way of treating optic nerve injuries.

Olfactory ensheathing cells (OECs) are unique glial cells that are responsible for the regeneration of olfactory axons throughout the life of adult mammals. They exist in the olfactory mucosa, olfactory nerve, and the two superficial layers of the olfactory bulb (the olfactory nerve fiber layer and olfactory glomerulus layer). Olfactory ensheathing cells have the same characteristics as astrocytes in the central nervous system and Schwann cells in the peripheral nervous system; however, they differ from other types of glial cells in terms of development, morphology, and immunohistochemistry [5,6]. It has been shown that OECs secrete neurotrophic factors (NTFs), including nerve growth factor (NGF), brain-derived neurotrophic factor (BDNF), neurotrophin-3/4 (NT-3/4), and ciliary neurotrophic factor (CNTF). These NTFs are related to the survival, differentiation, growth, and maturation of olfactory neurons, and they may serve as the major factors for promoting nerve growth, regeneration, and repair [7,8]. Following injury to the nervous system, glial scars impede the regeneration and elongation of nerve fibers. Olfactory ensheathing cells may penetrate glial scars and establish a glial tunnel, thus promoting nerve regeneration and repair [9,10]. Olfactory ensheathing cells also have the same characteristics as oligodendrocytes and neurons. It has been demonstrated that OECs promote the regeneration of central nerves and the spinal cord [11-13]. Therefore, OECs have attracted much attention from researchers in recent years and have been used in cell transplantation to treat central nervous system injuries. It has been shown that OECs implanted into an injured central nervous system formed cellular bridges that guided axon growth and prolongation [6,11].

Glial cell line-derived neurotrophic factor (GDNF) is one of the most potent neurotrophic factors that promote in vitro neuronal growth. It not only prevents neuron apoptosis during development, but also promotes the survival of cortical neurons following nerve injury [14]. Glial cell line-derived neurotrophic factor has been found to protect spinal motor neurons and peripheral nerves significantly in in vitro experiments and after sciatic nerve transection [15,16]. Therefore, we postulated that GDNF is important in the regeneration and functional recovery of the optic nerve after injury. Recombinant human glial cell line-derived neurotrophic factor (rhGDNF) is a 30 kDa homodimer consisting of two disulfide-linked 134 amino acid subunits. It is produced from recombinant DNA expressed in *E. coli*. Recombinant human glial cell line-derived neurotrophic factor can be obtained commercially and is suitable for future clinical use. In the present study, we injected rhGDNF into a vitreous body, cultured OECs, and transplanted them into injured optic nerves, then performed an intravitreous injection of wheat germ agglutinin (WGA) to anterogradely label retinal ganglion cells (RGCs), and used flash visual evoked potential (F-VEP) to assess the effect of OEC transplantation and GDNF intravitreal injection on the functional recovery of optic nerves after incomplete injury.

## Methods

### Culturing, purification, and transplantation of OECs

As described previously [17], the olfactory nerve layer and olfactory bulb granular layer were obtained from 60-day-old male Sprague-Dawley rats (SD rats; provided by the Experimental Animal Center of the Third Military Medical University, Chongqing China), and were digested with 0.1% trypsin. The digests were added to Dulbecco's Modified Eagle Medium Nutrient Mixture F-12 (DMEM/F-12; Gibco, Grand Island, NY) containing 10% fetal bovine serum (FBS; Gibco) and were mechanically dissociated into a single cell suspension. The cells were then inoculated into glass flasks not covered with gelatin. After the addition of DMEM/F-12 containing FBS, the flasks were cultured for 12 h in air containing 5% CO_2_ at 37 °C. The culture supernatants along with non-adhered cells were then transferred into another glass flask not covered with gelatin, followed by another 12 h of culturing. After that, the cell density was adjusted to 1×10^6^/ml and the cells were implanted into 6-well plates pretreated with polylysine, followed by another week of culturing. The cells were treated with Arabinofuranosylcytosine (final concentration, 10^−5^ mol/l Ara-C; Invitrogen, Carlsbad, CA) for 48 h, washed, and added to 2 μmol/l forskolin and 20 μg/ml bovine pituitary extract (BPE; Sigma, Carlsbad, CA). After approximately 10 days of culturing, the cells were transplanted and identified by immunofluorescence staining with anti-nerve growth factor receptor (NGFR-p75) antibody (diluted 1:50; Sigma,) to determine cell purity. Low Hoechst concentrations and appropriate durations of treatment can result in fluorescence labeling of cell nuclei without influencing cell viability. Most OECs used for transplantation were obtained by direct digestion (final concentration, 1×10^7^/ml). Some OECs were subjected to nucleus labeling with 10 μg/ml dibenzimide (Hoechst 33342; Sigma) through 30 min incubation at 37 °C. After labeling, the cells were washed twice with serum-free DMEM/F-12. The cell density was adjusted to 1×10^7^/ml before cell transplantation.

### Animal model preparation and groups

All procedures accorded with the Association for Research in Vision and Ophthalmology Resolution guidelines. The rats were maintained on food and water with a 12 h:12 h light-dark cycle (7:00 AM–7:00 PM). All operations were performed on animals anesthetized with intraperitoneal injections of ketamine (80 mg/kg) and xylazine (8 mg/kg). Before all operations, 0.4% oxybuprocaine hydrochloride (Alcon Laboratories, Fort Worth, TX) was applied to the eyes for superficial anesthesia and antiseptic eye drops (0.3% tobramycin, Alcon Laboratories, Inc.) were used to prevent post-treatment infection. All animals were humanely euthanized with an overdose of anesthesia. After anesthetization, the rats were placed in the lateral decubitus position. Under microscopy, the lateral canthus and the temporal ocular conjunctiva were cut open and subconjunctival and postbulbar tissues were isolated to expose the optic nerve. The optic nerve was clamped for 10 s using an atraumatic vascular clamp 1.5 mm behind the eyeball to cause moderate optic nerve injury (refer to the models of various degrees of incomplete injury to the rat optic nerve established previously). The ocular artery should not be injured. Antibiotic eye drops and ointments were used postoperatively. Only one eye (the right eye) was operated on. The blood supply of the rat ocular fundus was observed postoperatively and rats without fundus ischemia were included in the following experiments.

Eighty adult male SD rats (weighing 240–260 g) were randomized into four groups (20 animals in each group). The vitreous cavities of one group were injected with rhGDNF (GDNF group). Another group was transplanted with OECs in the injured optic nerve (OEC group). A third group was treated with an rhGDNF injection and OEC transplantation (GDNF+OEC group). In addition, because a previous experiment showed a balanced salt solution injection at the traumatized area was ineffective, a balanced salt solution was injected into the vitreous cavities of the fourth group (Control group). For each group, treatment occurred at five time points, i.e., immediately after injury and at 1, 2, 4, and 8 weeks after injury. For each group of 20 animals, five animals were assessed and then sacrificed at weeks 1, 2, 4, and 8 after injury.

### Intravitreal injection

A sterile microinjector was introduced obliquely backward and downward at the ambitus of the eyeball (3 mm from the sclerocorneal limbus) for the anesthetized rats. The needle tip was monitored directly through the pupil to avoid injuring the lens and 2 μl (2 μg) rhGDNF (G2781; Promega Corporation, Madison, WI) was injected into the vitreous cavity.

### Transplantation of OECs

After the optic nerve was exposed, 2 μl of an OEC suspension (cell concentration, 1×10^7^/ml) was transplanted into the proximal end 1 mm away from the injury site using the microinjector. The tip of the injector pricked the myelin sheath of the optic nerve and was inched toward the injury site for 0.5 mm. The OEC suspension was then injected slowly for more than 1 min and the needle was left in place for 2–3 min. The time points for injection were immediately after injury and 1, 2, 4, and 8 weeks after injury. The rhGDNF was prepared as described previously [15]. The purity of the rhGDNF was 96%.

### Visual electrophysiology investigation

Flash visual evoked potential (F-VEP) was performed on the eyes before injury, immediately after injury, and before sacrifice. The rats were anesthetized and immobilized on a special supporter. A ROLAND RETIport (Roland Consult, Brandenburg, Germany) visual electrophysiology system with silver needle electrodes was used. The recording electrode was inserted into the subperiosteum of the rat occipital tuberosity and the reference electrode was inserted subcutaneously at the midpoint between the two eyes. The ground electrode was inserted subcutaneously at the ear tip. Full visual field white flash stimulation was applied, with a flash intensity of 3. 93cd/m^2^s without background light and with a stimulation frequency of 2 Hz, a band pass width of 1–100 Hz, and at 20,000× magnification. The time for each sampling was 250 ms and the waveform was superimposed 50 times. Each rat was recorded three times with an interval of 10 min. The parameters observed were F-VEP latency LP1 (P1 wave response time, ms) and amplitude AP1-N2 (delta between P1 wave trough and N2 wave peak, μV). Three measurements were averaged for each parameter.

### Anterograde labeling with WGA for RGCs axons

Intravitreal injection of WGA (Sigma) was performed to anterogradely label RGC axons as described by Weibel [18]. The procedure was as follows: After anesthesia, 5 μl 2.5% WGA was injected into the vitreous bodies of the rats. Then at each experimental point, the rats were perfused with 4% paraform and the optic nerve was taken out and fixed in 4% paraform containing 20% sucrose at 4 °C for 10 h. The fixed optic nerve was then embedded by Optimal Cutting Temperature compound (OCT; SAKURA Inc., New York, NY) and cut into 10 μm sections. The primary antibody (containing 3% BSA, 0. 3% TritonX-100, and 0.01 mol/l PBS) goat-anti WGA (1:200) was added and incubated for one night at 4 °C, then washed three times. Secondary antibody rabbit-anti-goat fluorescein isothiocyanate (FITC; 1:64) was added and incubated for 2 h at 37 °C.

### Statistical analysis

Data were presented as mean±standard error of the mean (SEM). Statistical analysis was made using either a Student’s two-tailed *t*-test or ANOVA with SPSS11.0 software. Differences were considered to be significant at p<0.05 and statistically very significant at p<0. 01.

## Results

### Detecting the purity of OECs

**Figure 1:**
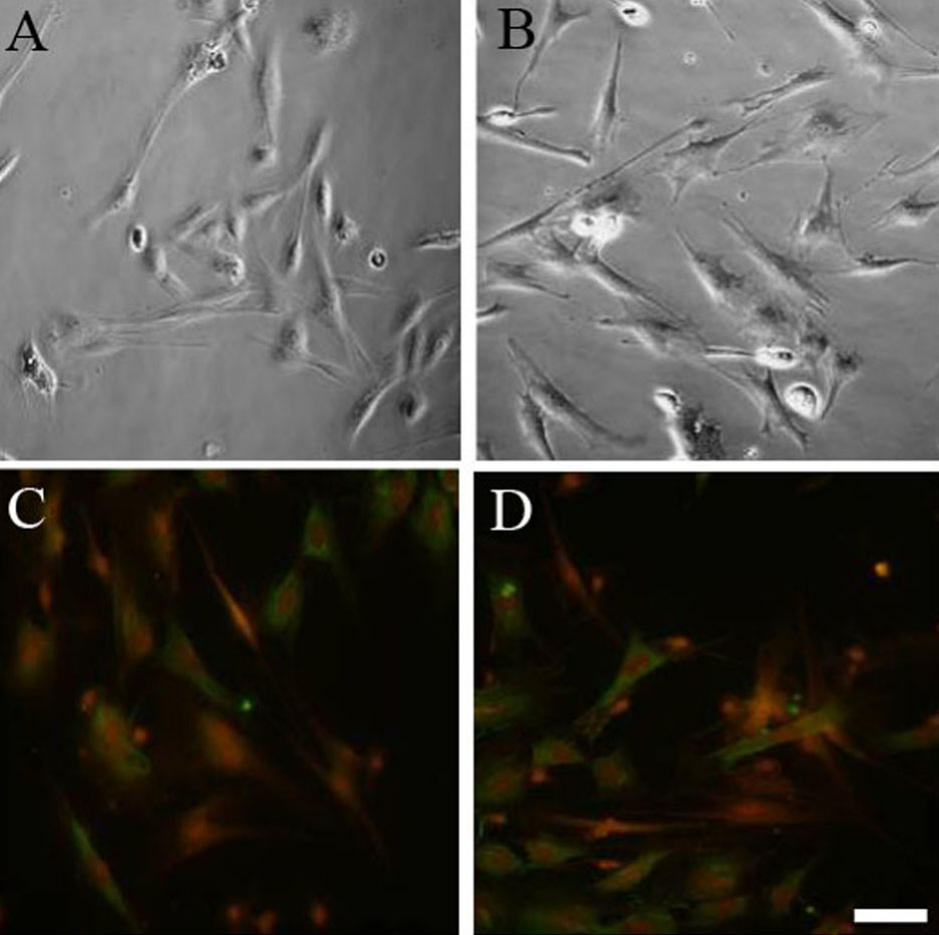
Olfactory ensheathing cells culture, purification, and immunofluorescence staining. **A**: OECs cultured for 8 days, **B**: OECs cultured for 14 days **C**: cultured for 8 days, **D**: cultured for 14 days OECs with immuofluorescence staining. NGFR-p75 staining for OEC (red); fibronectin staining for ONF (green). The scale bar is 20 μm.

The purified OECs were cultured for 6 days. Most cells displayed short processes with a three-dimensional cell body and good refractiveness. The cells were identified as OECs using immunofluorescence staining (Figure 1). After about 10 days of culturing, the purified cells were found to have long and thin processes, and the cells were in a bipolar or multipolar shape and were interconnected in a network. There were a small number of background cells, which had large cell bodies, poor three-dimensional sense, an irregular shape, and they formed a gossamer structure. These were fibroblasts. Based on purity calculation (the ratio of the number of immunohistochemically stained cells to the number of all cells), the cell purity was above 90%.

### Animals with intravitreal injection and/or OEC transplantation

Five rats from each group were examined at each time point (including immediately after injury and 1, 2, 4, and 8 weeks after injury). The rats were sacrificed after VEP investigation at each time point except immediately after injury; therefore, a total of 80 rats were used in this study. All the rats in the GDNF group that received intravitreal rhGDNF injections and Control group rats successfully endured the experiment. One rat in the OEC group died two days after OEC transplantation. One rat in the GDNF+OEC group died one day after the animal model preparation and treatment operation, and one rat died two days after F-VEP investigation at 4th week time point. Thus, in this experiment, 96.3% (77/80) of the rats survived the optic nerve injury model preparation, intravitreal injections, and cell transplantation.

### F-VEP investigation

**Figure 2:**
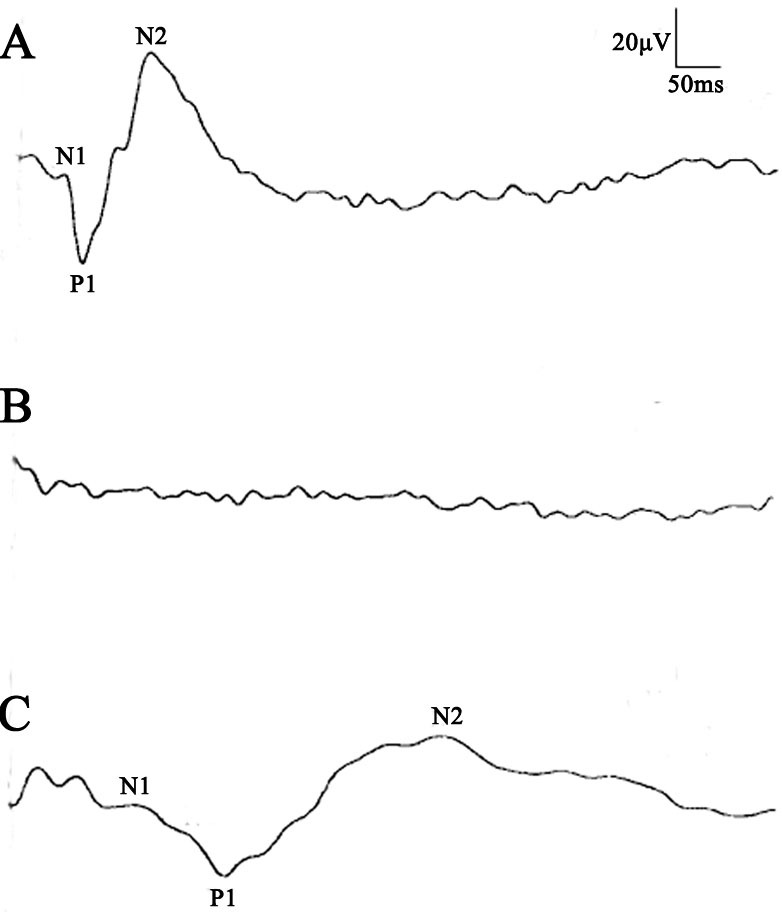
Representative waveforms of flash visual evoked potential (F-VEP) in rats. **A**: Each peak of F-VEP could be distinguished clearly in normal rats, including N1, P1, and N2 waves. **B**: Immediately after the optic nerve injury, the F-VEP waveform appeared silent and peaks could not be identified. **C**: Four weeks after OEC+GDNF treatment, F-VEP latency obviously decreased and amplitude increased significantly in rats with injured optic nerves.

**Figure 3:**
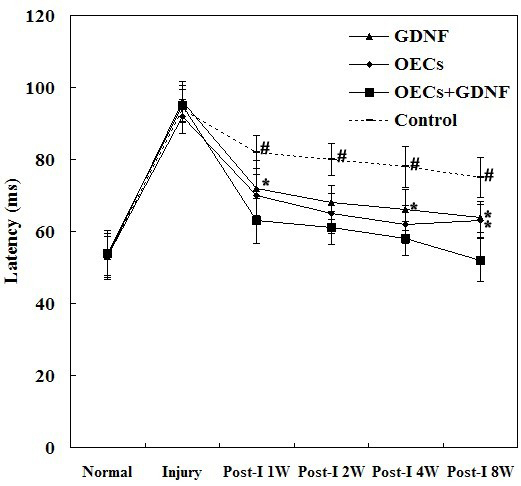
Latency (LP1) of flash visual evoked potential (F-VEP) before and after optic nerve injury. The LP1 was obviously delayed immediately after the optic nerve injury and was then rapidly restored 1 week after the injury, especially in the OEC+GDNF group. At 8 weeks, the LP1 of the OEC+GDNF group was almost restored to the normal value, but it was still obviously delayed in the Control, OEC, and GDNF groups. Data were shown as mean±SD (n=5). #: p<0.01 versus OEC+GDNF group; *: p<0.05 versus OEC+GDNF group.

**Figure 4:**
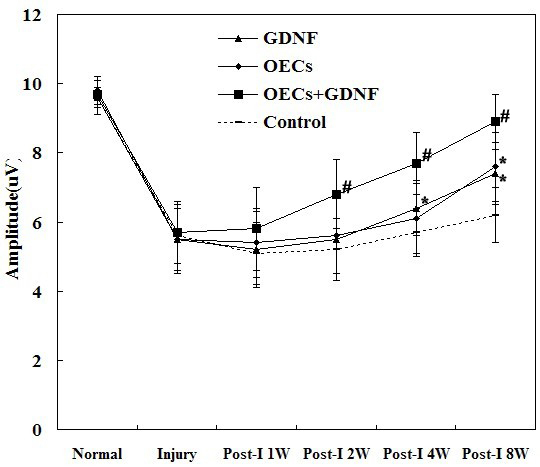
Amplitude (AP1-N2) of flash visual evoked potential (F-VEP) before and after optic nerve injury. The AP1-N2 obviously decreased immediately after the optic nerve injury, and was then restored very slowly. At 2 weeks, the AP1-N2 of the OEC+GDNF group were obviously restored compared with the Control, OEC, and GDNF groups. At 4 weeks and 8 weeks after injury, the AP1-N2 of the OEC+GDNF, OEC, and GDNF groups increased significantly compared to the Control group. #: p<0.01 versus Control group; *: p<0.05 versus Control group. Data were shown as mean±SD (n=5).

The F-VEP investigations were performed successfully using a ROLAND RETIport visual electrophysiology system and the waveforms could be clearly distinguished (Figure 2A). Immediately after the optic nerve injury, the F-VEP waveforms were almost silent, the latency was obviously prolonged, and the amplitude was significantly reduced (Figure 2B). At 1 week after injury, the latency was almost restored to the pre-injury level in the treatment groups, especially for the GDNF+OECs group (p<0.01 versus Control and p<0.05 versus GDNF groups; Figure 2C); it then decreased gradually (Figure 3). The amplitude was restored more slowly, and at 1–2 weeks after injury, the amplitude did not differ significantly between the treatment groups and the Control group (p>0. 05). At 4 weeks after injury, the OEC group differed significantly from the Control group (p<0. 05), and the GDNF+OECs group differed from the Control group very significantly (Figure 4). At 8 weeks after injury, the amplitude was restored to 64.5% of the pre-injury level in the Control group, to 77.1% in the GDNF treatment group, to 77.6% in the OEC treatment group, and to 91.8% in the GDNF+OECs treatment group.

### WGA labeling for RGC axons regeneration

**Figure 5:**
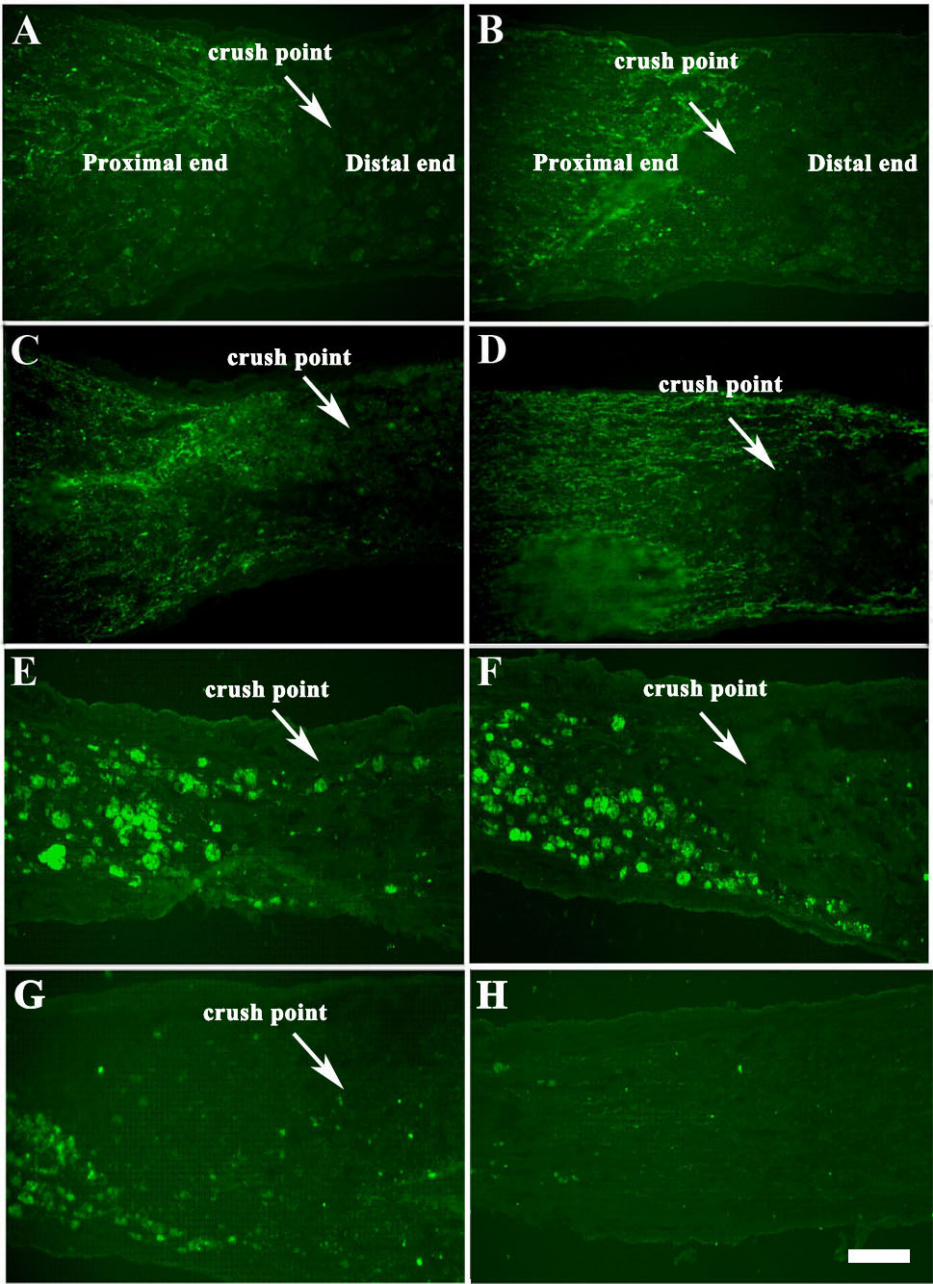
The longitudinal sections of RGC axons were labeled with wheat germ agglutinin (WGA) for axonal regeneration. **A**: After optic nerve injury, the ends of axons became accumulated and twisted. **B**: One week after injured rich WGA-labeled axons were visualized at the proximal end of the traumatized area, a few axons entered the distal end. **C**: One week after injury, the ends of the axons in the OEC+GDNF group were more extense and outspread. **D**: Two weeks after injury, some axons entered the traumatized area and reached the distal end of the traumatized area. **E**: Four weeks and **F**, 8 weeks after injury, WGA-labeled nerve fibers in the OEC+GDNF group were obviously prolongated and displayed abundant regenerated axons. At these time points the figure showed regenerated axons tangled and fluorescence accumulated there. **G**: Eight weeks after injury, a few new fibers regenerated in the OEC group and extended across the traumatized area to reach the distal end; however, the regenerated axons appeared significantly less than in the OEC+GDNF group. **H**: Negative control for WGA-labeled optic nerve axons. All images: left is the proximal end and right is distal end of optic nerve injured point. The scale bar is 100 μm.

The WGA labeled axons of normal optic nerves were longitudinally arrayed and appeared even and intensive. After the optic nerve injury, the ends of axons accumulated and became twisted, even appearing backward, and seldom entered the traumatized area (Figure 5A). After being injured for 1 week, rich WGA-labeled axons were displayed at the proximal end of the traumatized area and the density of WGA-labeled axons decreased significantly at the distal end about 500 μm away from the traumatic point; a few axons entered the distal end (Figure 5B). However, 1 week after injury, the ends of the axons in the GDNF+OEC group were more extensive and outspread (Figure 5C). At 2 weeks, some axons entered the traumatized area, especially the axons adjoined to the optic nerve sheath (Figure 5D). At 4 weeks (Figure 5E) and 8 weeks (Figure 5F) after injury, the WGA-labeled nerve fibers in the GDNF+OEC group were obviously prolongated and displayed abundant regenerated axons. Eight weeks after injury, some nerve fibers in the OEC group degenerated and a few new fibers regenerated and extended across the traumatized area and reached the distal end. However, the regenerated axons appeared significantly less than in the OEC+GDNF group (Figure 5G).

## Discussion

Optic nerve repair has long been the focus of research for many scholars. Breakthroughs in this field will shed light on central nerve repair after injury and regeneration. The optic nerve consists of RGC axons. Therefore, visual recovery is closely associated with the degree of RGC injury, the status of material transport and the synthesis of axoplasm, and the ability to self-repair and regenerate [19,20]. The F-VEP can objectively reflect the degree of optic nerve injury and nerve conduction; hence, it can be used to assess retina-visual cortex conduction. The F-VEP latency reflects the function of nerve conduction and axon myelin sheath integrity, and F-VEP amplitude reflects the receptive function of the macula lutea and the number of synaptic contacts between intact axons and their targets [21]. In the present study, we observed a nearly silent pattern of F-VEP waveforms immediately after injury, indicating a shock to visual function, which may be caused by increased pressure in the nerve tract, axon hypoxia, and transient blockage of nerve conduction due to nerve edema [22]. At 1 week after optic nerve injury, F-VEP latency was almost restored to the pre-injury level in the experimental groups, especially in the combined treatment group. The rapid recovery of the latency suggests that visual stimulation can cause cortical responses within a short time period if there are a certain number of intact optic nerve axon myelin sheaths and normal synaptic contacts in the target tissues after relief from the shock. Sabel [23] demonstrated that light perception can be restored if approximately 10% of RGCs had contact with target tissues. However, in clinical settings, vision can be partially restored in some patients with optic nerve injury without treatment [24]. This suggests that the optic nerve can repair itself to a certain extent after incomplete injury. At 4–8 weeks after injury, 60%–80% of F-VEP amplitude was restored to normal values, even to above 90% of the pre-injury level in the treatment group with subsidence of axonal edema, restoration of axoplasmic transport, and partial recovery of the axon regenerative ability. The recovery of F-VEP amplitude was significantly better in the treatment group than the Control group, suggesting that combined treatment promotes visual functional recovery. Incomplete and slow recovery of F-VEP amplitude may be related to the limited regenerative ability of the optic nerve after injury and progressive nerve tissue degeneration. Therefore, we believe F-VEP amplitude is a better parameter than F-VEP latency to reflect visual function in animal studies.

Glial cell line-derived neurotrophic factor has multiple biologic effects and is thought to play dual roles in neuronal survival and axonal regeneration [6]. Continuous injections of rhGDNF into the vitreous cavity may work by replacing neurotrophic factors or promoting the transportation of neurotrophic factors, enhancing the resistance of RGCs to primary injury (and particularly to secondary injury), and mitigating the secondary degeneration and apoptosis of neurons with minor injury or without injury, thus protecting RGCs and slowing down nerve tissue degeneration and preserving as many nerve fibers as possible. It may also work by enhancing nerve regeneration [25] and promoting the prolongation of regenerating axons after incomplete injury. It may also promote the recovery of axoplasmic transportation, enhancing synthesis and metabolism in nerve cells, regulating nerve nutrition, mitigating tissue edema, relieving topical pressure, and improving the internal environment of nerve tissues, thus helping the functional recovery of impaired nerve fibers.

In previous study, sole GDNF exhibited certain protective effect on neurons after spinal injury, but it did not promote axon regeneration significantly in this study. This may be explained as follows. First, GDNF promotes axonal regeneration, but regenerated axons cannot grow into the injured spine and scars. Second, GDNF is metabolized quickly after it is injected into the body. Olfactory ensheathing cells are cells that exist in the transitional zone of the central and peripheral nervous systems and are functionally active. They have become one of the preferred cell types in the treatment of spinal injuries in recent years. The present study demonstrated that central axons were regenerated after injury only in the two groups treated with OECs. In the present study, we found for the first time that axonal regeneration did not greatly differ between the GDNF and Control groups, but the combination of GDNF and OECs promoted the OEC repair of spinal injuries, suggesting the synergism of GDNF and OECs. Such synergism can be explained by the following mechanisms: (1) During the late stage of injury, glial scars secrete large amounts of inhibitory factors and act as a physical barrier to axon growth. As a result, GDNF cannot promote regenerating axons to penetrate glial scars. The OECs may serve as a cell bridge, guiding nerve fibers to grow and penetrate glial scars. Hence, OECs exhibited a synergistic action with GDNF in promoting axonal regeneration. (2) Glial cell line-derived neurotrophic factor may protect transplanted OECs and may even promote the proliferation of OECs, thus enhancing the action of OECs.

### Conclusion

We propose that OEC transplantation and rhGDNF injection can promote the recovery of visual function after the incomplete injury of the optic nerve in adult rats and that a combination of OECs and rhGDNF will be most effective. The present study sheds light on the regeneration and repair of the optic nerve and even central nerves following injury and provides a new treatment option with certain clinical significance.
